# EuraHS: the development of an international online platform for registration and outcome measurement of ventral abdominal wall hernia repair

**DOI:** 10.1007/s10029-012-0912-7

**Published:** 2012-04-18

**Authors:** F. Muysoms, G. Campanelli, G. G. Champault, A. C. DeBeaux, U. A. Dietz, J. Jeekel, U. Klinge, F. Köckerling, V. Mandala, A. Montgomery, S. Morales Conde, F. Puppe, R. K. J. Simmermacher, M. Śmietański, M. Miserez

**Affiliations:** 1 Department of Surgery, AZ Maria Middelares, Kortrijksesteenweg 1026, 9000 Ghent, Belgium; 2 Department of Surgical Sciences, Multimedica Hospital, University of Insubria-Varese, Castellanza, Italy; 3 Chef du Service de Chirurgie, Centre Hospitalo-universitaire Jean Verdier Digestive, Université Paris XIII, Avenue du 14 Juillet, 93140 Bondy, France; 4 Department of General Surgery, The Royal Infirmary of Edinburgh, 51 Little France Crescent, Edinburgh, EH16 4SA Scotland, UK; 5 Department of General, Visceral, Vascular and Pediatric Surgery (Department of Surgery I), University of Wuerzburg, Oberduerrbacher Strasse 6, 97080 Wuerzburg, Germany; 6 Department of Surgery, Erasmus Medical Center, ‘s Gravendijkwal 230, 3051 CE Rotterdam, The Netherlands; 7 Department of Surgery, University Hospital of the RWTH Aachen University, Pauwelsstraße 30, 52074 Aachen, Germany; 8 Department of Surgery, Vivantes Hospital Spandau, Neue Bergstrasse 6, 13585 Berlin, Germany; 9 Department of General and Emergency Surgery, United Hospitals Villa Sofia- Cervello, 90015 Palermo, Italy; 10 Department of Surgery, Malmö University Hospital, 20502 Malmö, Sweden; 11 Unit of Innovation in Minimally Invasive Surgery, University Hospital Virgen del Rocío, Betis-65, 1°, 41010 Seville, Spain; 12 Fakultät für Mathematik und Informatik Lehrstuhl für Künstliche Intelligenz und Angewandte Informatik (Informatik VI) Am Hubland, Universität Würzburg, 97074 Würzburg, Germany; 13 Department of Surgery, University Hospital, 3584 CX Utrecht, The Netherlands; 14 Department of General and Endocrine Surgery and Transplantation, Medical University of Gdańsk, 7 Debinki Street, 80-211 Gdańsk, Poland; 15 Department of Abdominal Surgery, Universitair Ziekenhuis Leuven, Herestraat 49, 3000 Leuven, Belgium

**Keywords:** Ventral hernia, Incisional hernia, Umbilical hernia, Epigastric hernia, Registries, Quality of life

## Abstract

**Background:**

Although the repair of ventral abdominal wall hernias is one of the most commonly performed operations, many aspects of their treatment are still under debate or poorly studied. In addition, there is a lack of good definitions and classifications that make the evaluation of studies and meta-analyses in this field of surgery difficult.

**Materials and methods:**

Under the auspices of the board of the European Hernia Society and following the previously published classifications on inguinal and on ventral hernias, a working group was formed to create an online platform for registration and outcome measurement of operations for ventral abdominal wall hernias. Development of such a registry involved reaching agreement about clear definitions and classifications on patient variables, surgical procedures and mesh materials used, as well as outcome parameters. The EuraHS working group (European registry for abdominal wall hernias) comprised of a multinational European expert panel with specific interest in abdominal wall hernias. Over five working group meetings, consensus was reached on definitions for the data to be recorded in the registry.

**Results:**

A set of well-described definitions was made. The previously reported EHS classifications of hernias will be used. Risk factors for recurrences and co-morbidities of patients were listed. A new severity of comorbidity score was defined. Post-operative complications were classified according to existing classifications as described for other fields of surgery. A new 3-dimensional numerical quality-of-life score, EuraHS-QoL score, was defined. An online platform is created based on the definitions and classifications, which can be used by individual surgeons, surgical teams or for multicentre studies. A EuraHS website is constructed with easy access to all the definitions, classifications and results from the database.

**Conclusion:**

An online platform for registration and outcome measurement of abdominal wall hernia repairs with clear definitions and classifications is offered to the surgical community. It is hoped that this registry could lead to better evidence-based guidelines for treatment of abdominal wall hernias based on hernia variables, patient variables, available hernia repair materials and techniques.

## Introduction

Randomised clinical trials (RCT) remain the source of the best evidence. However, in a RCT, the randomised controlled variable is just one out of many. The long delay from surgery to the development of many complications such as recurrence and the impossibility to control all relevant parameters can hinder proof of the significant impact, in particular, when studying slight modifications of techniques or materials. For this reason, the alternative second choice is a registry. This allows the detection of poor and good results, if they appear more frequently than expected. National Scandinavian registries, like the Swedish Hernia Database and the Danish Hernia Database on hernia surgery, have demonstrated this [1–4]. Also multicentre databases like the Veterans Affairs Medical Centers database and the National Surgical Quality Improvement Program database have been able to detect poor outcome results in hernia surgery [5–7].

During the 4th International Hernia Congress in Berlin in 2009, a working group was formed under the auspices of the European Hernia Society board, with the task of developing a registry for operations on abdominal wall hernias. The project was named EuraHS (European Registry for Abdominal Wall HerniaS). The EuraHS working group was formed by the first author with a panel of surgeons from different European countries, who have a known interest in hernia surgery and research. Five working group meetings were organised to reach a consensus on a clear description of the scope of the registry and the data to be collected in the registry.^1^

The mission of the EuraHS working group is to provide an international online platform for registration and outcome measurement of hernia operations, which includes a set of definitions and classifications for use in clinical research on abdominal wall hernias.

## Materials and methods

**Fig. 1:**
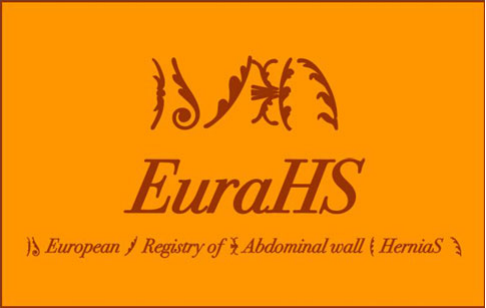
Logo of EuraHS: European registry of abdominal wall hernias

A EuraHS logo is agreed upon and a website http:\\www.eurahs.eu is provided (Fig. 1). Access to the database will be through the website. The website will contain all the classifications and definitions as proposed by the EuraHS working group. Important papers and guidelines, as well as the reports from the database will be downloadable from the website. The IT platform for EuraHS is developed at the department of Artificial Intelligence and Applied Informatics, part of the Institute for Mathematics and Computer Science, at the University of Würzburg in Germany, under the supervision of Prof Dr Frank Puppe. From January 2012 till May 2012, a test phase on the performance of the EuraHS platform by the working group members is conducted. The EuraHS platform will be available for the surgical community as of 7 June 2012, when the platform will be launched during the EuraHS Launch Symposium.

### A consensus model

The EuraHS working group decided on the variables to be included in the database. Existing classifications were used where possible, but many variables needed new descriptions, definitions and classifications. These were formed by consensus between the working group members from nine different European countries.

### Scope of the database

The scope of the EuraHS registry will be primary ventral hernias, incisional ventral hernias and parastomal hernias in adult patients older than 18 years. Hernia operations and not patients will be registered. A patient who is operated a second will be recorded as a new case. An attempt will be made to convince existing European hernia databases, to join the EuraHS and to collect their data on the same Internet platform.

The database will be used on a voluntary basis. A stratification of users will be offered. A *Level 1 user* will only have a small number of compulsory data fields to complete the registration of a case. These data will involve the variables needed for classification of the hernia, the surgical technique used and the materials used during the repair. Uploading a case should only take a few minutes. A *Level 2 user* will have the availability to complete a more comprehensive number of variables for surgeons with a specific interest in hernia surgery. This level is designed for surgeons or groups of surgeons who will collect the data set as complete as possible and who commit themselves to a follow-up of many years.

### Ownership of the data

The surgeon uploading a case using his or her account will be the owner of the data. The user will be able to retrieve their data at any time in Excel files. Moreover, a standardised set of tables and figures with the users data will be available and downloadable.

Data can be shared in groups. A surgeon can decide to group their data with the data of other surgeons within the same hospital and therefore will be able to retrieve the overall data of the institution. Every user will be asked whether the institutional data can be shared amongst the members of the institution.

Multicentre groups can be formed. When uploading a case, a possibility will exist to upload this case into a multi-user group, with a specific name and password. The users can retrieve the specific data of the group. This will allow surgeons performing multicentre and even international trials to collect their data easily with a standardised set of data.

In every country where surgeons contribute cases to the EuraHS database, one or more national EuraHS representatives will be appointed. The national representatives will perform access control to the EuraHS. When making a new account, a user will need acknowledgement by a national representative to enter the database. The national representative will be able to extract the national overall data, anonymous for patients and surgeons.

The EuraHS working group will have access to all of the anonymous data held on the EuraHS database. This will allow an annual report to be published on the EuraHS website.

Acknowledgement of the EuraHS database as the source of the data has to be made every time it is used in public or in publications.

### Quality of the data

The registry will not contain personal data like names or date of birth and will thus be completely anonymous. The link between the EuraHS registration number and the patients’ identity will be the responsibility of the user. Tools with sets of data will be made available to track the patients’ identity if the users lose the link between the EuraHS registration number and the patient identity.

The users of the database will be responsible for the quality of their data. All Level 1 data will be needed to complete a registration. The quality of the follow-up data will depend on the commitment of the users to perform the follow-up and upload the data. Tools will be made available to alert the users at specific follow-up time points if they choose to get these reminders.

### Informatics and mathematics solutions for the database

The quality of EuraHS database and the dialogue^2^ will have a huge impact on the success of our voluntary database. It is important that their quality equals the performance of other online applications we use in our daily life.

**Fig. 2:**
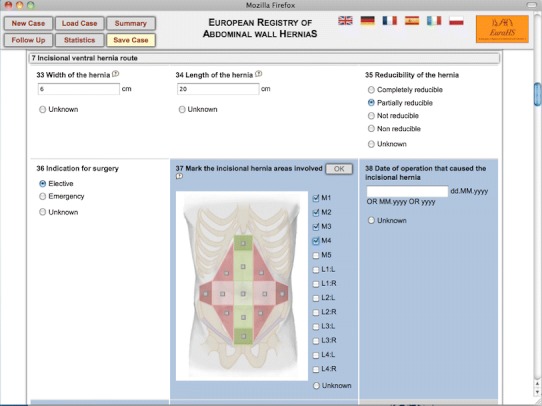
Screenshot of the dialogue for data input into the EuraHS database. A *blue background* of a question indicates that it has not been answered yet

The technical requirements for the dialogue to input data in the database are complex, including a multilingual database, a compact layout and a fast reload time. To avoid too many simultaneous questions on the computer screen, the database will contain follow-up questions only showing when relevant (Fig. 2). The database will include image questions, where the answers are given by clicking on an area of the image. When needed “pop-up” boxes with key definitions of the variables will be available on demand. Some automatic computations like BMI from weight and height of the patient will be available. The materials used during surgery will be selected from alphabetic “drop-down” boxes.

The terminology of the database and the additional knowledge are entered with the semantic wiki KnowWe, from which the dialogue is generated with a dialogue prototyping tool allowing experimentation with different dialogue designs [8, 9].

The cases are stored in a database from which various statistical analyses can be started from the web interface (button “statistics”). The users will be able to extract their data in tables and in diagrams. The quality of this return data to the users will be the most important incentive for users to continue using the database.

## Results

**Fig. 3:**
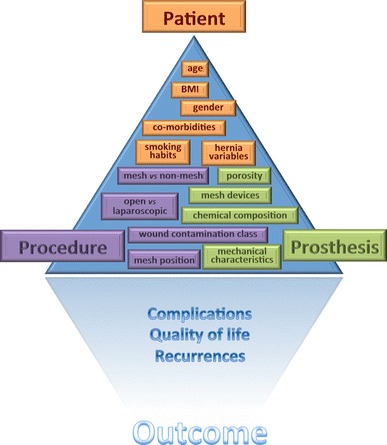
The triple-P triangle of abdominal wall hernia repair

A comprehensive database on abdominal wall surgery can only be built if based on a clear set of definitions and classifications on the three *P*-*entities* involved in these operations: *Patient*-*Procedure*-*Prosthesis* (Fig. 3). The outcome of operations will depend on the interaction between these three entities and their different variables that all might have influence on the outcome. It is this large number of variables in each P-entity that can make evaluation of abdominal wall hernia repairs so difficult. Definitions and a clear nomenclature of the variables are essential. Definitions and classifications on the outcome parameters were also needed to allow a coherent description of the results.

### Patient entity

One goal of the registry is to detect patient variables that are of importance for the outcome parameters: complications, recurrences and quality of life. Some patient variables are straightforward like age, gender, BMI. Other variables like the hernia characteristics and patient co-morbidities need specific definitions and classifications.

#### Definitions of abdominal wall hernias

Table 1 gives the EuraHS proposal of definitions for different ventral hernias. Inguinal hernias definitions have already been proposed in the EHS groyne hernia classification and the EHS groyne hernia guidelines [10, 11]. The proposed terminology being: medial inguinal, lateral inguinal and femoral hernias.

**Table 1:** EuraHS definitions of ventral abdominal wall hernias

The abdominal wall	The *abdominal wall* is the musculo-fibrous covering of the abdomen containing the abdominal contents
Abdominal wall hernia	An *abdominal wall hernia* is an abnormal protrusion of the contents of the abdominal cavity or of pre-peritoneal fat through a defect or weakness in the abdominal wall
Ventral hernia	A *ventral hernia* is a hernia of the abdominal wall excluding the inguinal area, the pelvic area and the diaphragm
Primary ventral hernia	A *primary ventral hernia* is a ventral hernia that was present at birth or that developed spontaneously without trauma to the abdominal wall as the cause of the hernia
Umbilical hernia	A primary ventral hernia with its centre at the umbilicus
Epigastric hernia	A primary ventral hernia close to the midline with its centre above the umbilicus
Spighelian hernia	A primary ventral hernia in the area of the fascia Spigelian aponeurosis
Lumbar hernia	A primary ventral hernia in the lumbar area
Secondary ventral hernia	A secondary ventral hernia is a ventral hernia that developed after a traumatic breach of the integrity of the abdominal wall
Incisional ventral hernia	A ventral hernia that developed after surgical trauma to the abdominal wall, including recurrences after repair of primary ventral hernias
Traumatic ventral hernia	A ventral hernia that developed after non-surgical penetrating or blunt trauma to the abdominal wall
Acute post-operative ventral hernia	An incisional hernia resulting from an abdominal wall dehiscence, either complete (with skin dehiscence) or incomplete (covered with intact skin) within 30 days after the operation
Parastomal hernia	An incisional hernia through the abdominal wall defect created during placement of a colostomy, ileostomy or ileal conduit stoma

#### Abdominal wall hernia classification

**Fig. 4:**
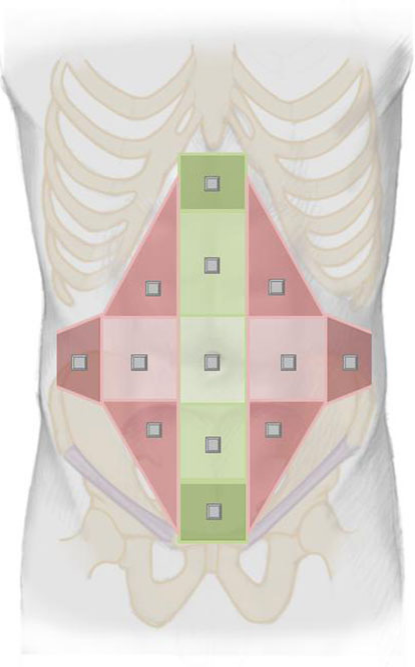
EuraHS ventral hernia model for registration and classification of abdominal wall hernias based on the localisation of the hernia

The previously described EHS classification of primary and incisional abdominal wall hernias will be used [12]. The user will indicate on a picture the abdominal wall areas that are involved (Fig. 4). The user of the registry will be asked to give the width and the length of the hernia according to the definition that will be shown in the dialogue with a “pop-up”. An intra-operative measurement of width and length is preferred above preoperative measurement clinically or with medical imaging. The database will provide the hernia classification automatically.

#### The SOC score: a severity classification of patient co-morbidities

Co-morbidity is generally considered to be an important risk factor for an unfavourable outcome. The American Society for Anaesthesiology Physical Status Classification System, better known as the ASA score, is widely used [13]. An increased ASA score correlates with an increased risk of operative morbidity and mortality. But ASA is not disease specific and will not allow the correlation of specific co-morbidities with an increased risk of unfavourable outcome in hernia operations. Therefore, the EuraHS database will include a novel severity classification of co-morbidities. This classification was named *SOC* *score or Severity Of Co*-*morbidity*-*score,* and the definitions are listed in Table 2. Validation of this SOC score will be one of the goals of the registry.

**Table 2:** EuraHS SOC score: a severity of co-morbidity scoring

Severity of co-morbidity score SOC score	
SOC score	Definition
0	No co-morbidities
1	Asymptomatic, no medical consultation needed in last 12 months
2	Stable disease, intermittent therapy and medical consultation needed ≤4x/year
3	Stable disease, continuous therapy with regular medical consultation >4x/year
4	Progressive disease, with changing or intensified therapy and frequent medical consultation >12x/year

Smoking has been found in several studies to be an important risk factor for the development of incisional hernias or of recurrences after hernia repair [14, 15]. In addition, for this risk factor, a gradation is needed, taking into account the amount of tobacco used.

### Procedure entity

Many different surgical options are available for the repair of abdominal wall hernias [16]. For most types of hernias, there is no widespread evidence-based consensus on the best treatment option. The type of surgical access, the use of mesh and the position of the mesh in relation to the abdominal wall will differ amongst these options.

#### Definitions of surgical techniques and mesh positions

The EuraHS database will capture the type of access to treat the hernia as open or laparoscopic surgery. In the laparoscopic group, there will be a subgroup for “conversions from laparoscopy to open surgery”. The number of trocars used during laparoscopic surgery will be captured making it possible to identify the number of single-port operations. Operations will be registered as either mesh repair or non-mesh repairs.

**Fig. 5:**
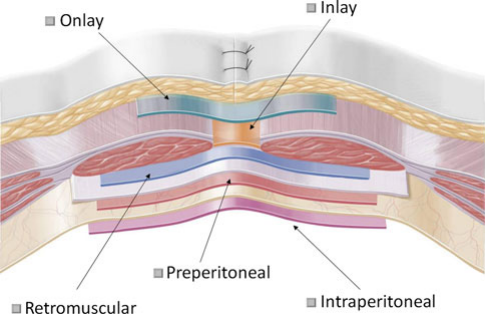
EuraHS terminology of mesh positions during ventral hernia repair

There is very little coherence on terminology for mesh positions across the globe. “Sublay” is used for a retromuscular position but also for intraperitoneal or preperitoneal. “IPOM or intraperitoneal onlay mesh” is used frequently in Europe but not in the USA. “Inlay” is either a position of the mesh inside the defect or an intraperitoneal mesh. “Overlay” is used as terminology in the USA for a premuscular position, while in Europe we call this an “Onlay” repair. To end this confusion, the EuraHS working group proposes the terminology as defined in Table 3 and illustrated in Fig. 5 [17, 18]. The choices in the database will be limited to these 5 options. Sometimes more than one mesh is used during operations or sometimes a mesh is placed in different positions in a patient. For these cases, a separate box will be available as “combined positioning”.

**Table 3:** EuraHS definitions of mesh position in ventral hernia repair

Onlay	The *onlay position* if the mesh is positioned above the abdominal wall muscles and fascia, behind the subcutaneous fat
Inlay	The *inlay position* if the mesh is positioned in the hernia defect, without overlap, and fixed to the margins of the defect
*Retromuscular*	
Medial hernias	The *retromuscular position for* *medial abdominal wall hernias* if the mesh is positioned behind the rectus abdominis muscle and in front of the posterior rectus fascia or -caudal to the linea arcuata- in front of the peritoneum
*Retromuscular*	
Lateral hernias	The *retromuscular position for lateral abdominal wall hernias* if the mesh is placed in a plane between the lateral abdominal wall muscles
Preperitoneal	The *preperitoneal position* if the mesh is placed in the plane behind all abdominal wall muscles in front of the peritoneum
Intraperitoneal	The *intraperitoneal position* if the mesh is placed behind all layers of the abdominal wall including the parietal peritoneum

Surgical techniques can also be described considering the handling of the hernia defect during the operation. In a *mesh augmentation technique,* the anterior fascia of the hernia defect is closed. In a *mesh bridging technique,* the anterior fascia of the hernia defect is not completely closed.

#### Grading of intraoperative contamination

The degree of intraoperative contamination during the hernia repair is considered to be an important variable. The Centre for Disease Control (CDC) classification of wound contamination will be used [19]. This classification scheme has shown in numerous studies to predict wound infection rate. The CDC classification and some examples for abdominal wall hernia repair are given in Table 4.

**Table 4:** CDC (centre for disease control) classification of wound contamination and examples for surgery in abdominal wall hernia repair [19]

Class of operation and wound contamination	CDC definition	Example for abdominal wall hernia repair
*Class I*: Clean	These are uninfected operative wounds in which no inflammation is encountered and the respiratory, alimentary, genital, or uninfected urinary tracts are not entered	Elective repair of a hernia
*Class II*: Clean-contaminated	These are operative wounds in which the respiratory, alimentary, genital, or urinary tract is entered under controlled conditions and without unusual contamination	Bowel lesion during adhesiolysis, without gross spillage of bowel content Combined cholecystectomy and hernia repair Bowel resection for incarceration Presence of a colostomy
*Class III*: Contaminated	These include open, fresh, accidental wounds, operations with major breaks in sterile technique or gross spillage from the gastrointestinal tract, and incisions in which acute, nonpurulent inflammation is encountered	Bowel lesion with gross spillage Enterocutaneous fistula
*Class IV*: Dirty	These include old traumatic wounds with retained devitalised tissue and those that involve existing clinical infection or perforated viscera. This definition suggests that the organisms causing post-operative infection were present in the operative field before the operation	Perforation of strangulated bowel Presence of infected mesh

### Prosthesis entity

Mesh repair is a Grade A recommendation for the treatment of inguinal hernias in adults given by the EHS guidelines [11]. There are no existing guidelines for incisional hernias, but the use of mesh is generally accepted for reinforcement of the abdominal wall during repair [20, 21]. The high number of hernia operations and thus the need for meshes has created a highly competitive market for meshes. Innovations and research on new mesh materials and mesh designs have provided us with a variety of choices. Moreover, several innovative mesh fixation devices with different forms and components, sometimes absorbable, have been introduced on the market.

The EuraHS will use the new classification of meshes described by Klinge et al. to group the meshes for use in the analysis of the data from the registry [22]. The EuraHS database will register the meshes, fixation devices, sutures and glues used during the operation with the product name. We cannot expect the surgeons to describe the chemical features of the product (polypropylene, polyester, ePTFE, PVDF, composite meshes, etc.) or the physical features of the product (weight, porosity, etc.). The development of the EuraHS platform will thus necessitate the construction of a comprehensive list of all the available mesh products, fixation devices, glues and sutures on the European Market. This listing will be available for all at the EuraHS website and a continuous updating of the list will be needed.

### Assessment of outcome: complications and recurrences

Complications can be defined according to the time of their occurrence in relation to the operation. Intra-operative complications, early post-operative complications, operative mortality, operative morbidity and late complications are defined in Table 5.

**Table 5:** EuraHS definitions of complications, morbidity and mortality

Intra-operative complications	Are complications occurring during the time of the patients’ arrival in the operating room and the patient leaving the operating room
“Acute” or “early” post-operative complications	Are complications occurring during the hospitalisation or within 30 days postoperatively
Late post-operative complications	Are complications related to the hernia repair occurring after discharge and more than 30 days postoperatively
Operative morbidity	The percentage of patients treated who had at least one complication occurring during the operation, during the hospitalisation or 30 days postoperatively
Operative mortality	The percentage of patients treated who died during the operation, during the hospitalisation or within 30 days postoperatively

#### Classification of early post-operative complications

Early post-operative complications are defined as complications occurring within 30 days postoperatively or before discharge (if longer than 30 days). The EuraHS database will use the Clavien-Dindo classification for grading the severity of post-operative complications as shown in Table 6 [23]. We have made a slight modification of the Clavien-Dindo classification by qualifying a puncture of a seroma as grade I, rather than it being a grade IIIa complication. When registering complications in the EuraHS database, this classification will be completed by responding to queries that will automatically be linked to a grade of complication. In patients with multiple complications, the patient will be graded with the complication having the highest grade.

**Table 6:** Clavien-Dindo classification and grading of post-operative complications [23]

*Grade 0*
No complications
*Grade I*
Any deviation from the normal post-operative course without the need for pharmacological treatment or surgical, endoscopic and radiological interventions (are allowed: antiemetica, antipyretica, analgetics, diuretics, electrolytes and physiotherapy. This grade includes wound infections opened at the bedside *and a seroma requiring aspiration bedside*.)
*Grade II*
Requiring pharmacological treatment with drugs other than such allowed for grade I complications. Blood transfusion and TPN are included.
*Grade III*
Requiring surgical, endoscopic and radiological interventions
IIIa Intervention not under general anaesthesia
IIIb Intervention under general anaesthesia
*Grade IV*
Life threatening complication requiring IC/ICU management
IVa Single organ dysfunction
IVb Multiorgan dysfunction
*Grade V*
Death of the patient

#### Late post-operative complications and recurrences

Late post-operative complications are defined as complications related to the hernia repair occurring after discharge of the patient and more than 30 days postoperatively. A recurrent abdominal wall hernia is a late negative event and is reported as a separate outcome measurement. We defined a hernia recurrence as follows: *A protrusion of the contents of the abdominal cavity or preperitoneal fat through a defect in the abdominal wall at the site of a previous repair of an abdominal wall hernia.* In the EuraHS database, users will be asked to postulate the cause for the recurrence. More than one cause can be chosen.

*Post-operative seroma* is a frequent event after repair of abdominal wall hernias. Some surgeons even consider it to be present in nearly every case. It usually resorbs and is often considered to be part of the normal post-operative course. Morales et al. have proposed a classification for post-operative seroma after laparoscopic surgery [24]. We will use it in the EuraHS database for open and laparoscopic operations. This classification can be found in Table 7 and is based on clinical findings and the presence of seroma-related complications.

**Table 7:** Classification of post-operative seroma after ventral hernia repair [24]

Type of seroma	Definition	Clinical significance
0	No clinical seroma	No clinical seroma
I	Clinical seroma lasting < 1 month	Incident
II	Clinical seroma lasting > 1 month	
III	Symptomatic seroma that may need medical treatment: minor seroma-related complications	Complication
IV	Seroma that need to be treated: major seroma-related complications	

*Clinical seroma:* Those seromas detected during physical examination of patients which do not cause any problem, or just a minimum discomfort that allows normal activity

*Minor complication:* Important discomfort which does not allow normal activity to the patient, pain, superfitial infection with cellulitis, aesthetic complaints of the patient due to seroma or seroma lasting more than 6 months

*Major complication:* Infection, recurrence, mesh rejection or need to be punctured

Another difficult issue is the *post-operative bulging* or so called pseudo-recurrence [25, 26]. If a surgical correction of the bulging is performed for cosmetic or symptomatic reasons, it will be considered a late complication.

*Chronic post-operative pain* is defined as pain present more than 3 months after surgery [27]. A verbal rating scale and classification of chronic pain has been published previously by Cunningham et al. and will be used in the EuraHS database [28]. Four grades are defined as follows: no pain, mild pain, moderate pain and severe pain (Table 8).

**Table 8:** Classification of chronic post-operative pain persisting 3 months after surgery [28]

Pain class	Definition
No pain	No discomfort experienced
Mild pain	Was defined to the patient as an occasional pain or discomfort that did not limit activity, with a return to prehernia lifestyle
Moderate pain	Was defined as pain preventing return to normal preoperative activities (i.e. inability to continue with prehernia activities such as golf, tennis and other sports, and inability to lift objects, without pain, that patient had been lifting before the hernia occurrence)
Severe pain	Pain that incapacitated the patient at frequent intervals or interfered with activities of daily living (i.e. pain constantly present or intermittently present but so severe as to impair normal activities, such as walking)

### Assessment of outcome: quality-of-life assessment

Several quality-of-life scores (QOL) have been used after surgery. Short Form 36 (SF 36) is a validated QOL assessment tool for surgery in general, but for QOL evaluation after hernia repair and specifically after mesh implantation, it has not been so useful [6, 29]. A QOL score specifically targeting patients that had an abdominal wall hernia repair with a mesh has been developed by Heniford et al. at the Carolina Hernia Centre in Charlotte, NC, USA [30]. This Quality-of-Life scale is commonly referred to as the *Carolina Comfort Scale* (*CCS*). The CCS holds a trademark, and thus, use of the CCS requires a licence agreement. Therefore, it cannot be integrated in our open access and free-for-all online platform.

**Fig. 6:**
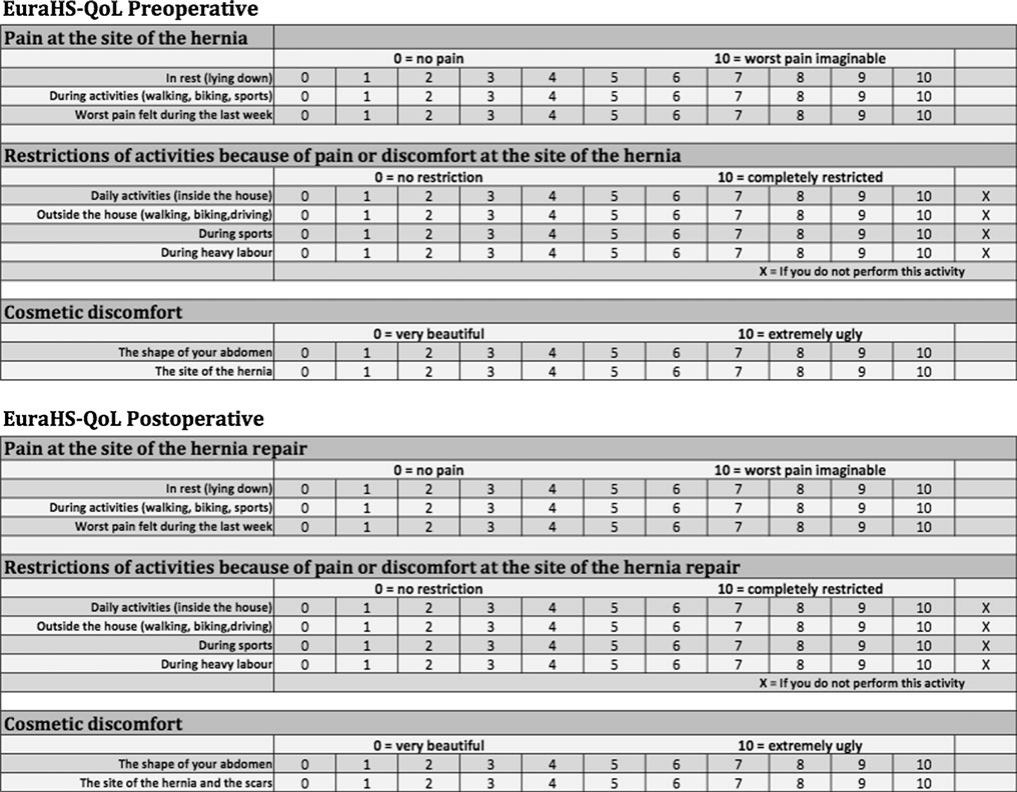
EuraHS quality-of-life score for pre- and post-operative assessment of patients with ventral abdominal wall hernias: EuraHS-QoL

The EuraHS working group proposed a “EuraHS-QoL” score for evaluation of QOL before and after ventral hernia repair and this is shown in Fig. 6. The score can be used for mesh and non-mesh repairs and is based on a Numerical Rating Scale for three dimensions: pain at the site of the hernia or the hernia repair, restriction of activities and cosmetic discomfort. The EuraHS-QoL adds some interesting features compared with other QOL scores, in particular, assessment made pre- and postoperatively and by including a cosmetic dimension which is an important but understudied element in ventral hernia repair. Validation of the EuraHS-QoL score will be part of the research by the EuraHS working group following the launch of the platform.

## Discussion

The European Hernia Society was founded in 1979 as the Grepa (Groupe pour la recherche sur la paroi abdominal) and took its current name in 1998. The aim of the society is as follows: *The promotion of abdominal wall surgery, the study of anatomic, physiologic and therapeutic problems related to the pathology of the abdominal wall, the creation of associated groups which will promote research and teaching in this field, and the development of interdisciplinary relations* [31].

A classification and guidelines for groyne hernia were developed and published [10, 11]. For primary and incisional ventral hernias, a classification was proposed [12]. The level of evidence currently available makes it impossible to provide guidelines and EBM recommendations of level A on most of the topics concerning ventral hernia repair. The EuraHS working group was created to provide for the surgical community an online database to collect the data and the outcome of their patients.

The concept and the approach to the development of the EuraHS database is guided by “the four rules of the New Normal” as described by Peter Hinssen is his book on how to have success in a digitalised world [32]. The EuraHS database has to be up-to-date and in line with what is available in other IT services in our life. The database should be easy to use and quick. Although one of the main goals of the EuraHS is to allow individual surgeons to collect their data in a standardised manner, it will be the user who will decide how detailed their contribution to the database will be. The incentive for the surgeon to contribute to the EuraHS database will be the quality of the database and the direct access to their own data. One or several of the users at their own initiative can form research groups. They will be able to extract their data and use it for presentations and publications. It will be a dynamic process. It is hoped that this platform and database will lower the threshold for the individuals to perform prospective studies.

Post-operative complications are an important outcome parameter to be recorded, but it is difficult to compare the results from different studies in the literature because they usually lack a description of the severity of the complications. Dindo et al. have written extensively on the grading of post-operative complications [23]. This is usually referred to as the “Clavien-Dindo classification” and is used in many other fields of surgery to grade the severity of a complication rather than only stating a percentage of patients that had a complication. Kaafarani et al. validated this classification for ventral hernia repair [33]. In a follow-up paper by Dindo et al., they reported on the difficulty of registration of post-operative complications [34]. The surgical residents, compared to the registration by a specially trained study nurse, did not record around 80 % of post-operative negative events. Indeed the Grade I—*any deviation from the normal post-operative course*—is depending of what the observer considers a normal post-operative course. Therefore, Grade I and Grade II will be underestimated, whereas Grade III–V will be more accurate. Considering this, data on post-operative complications gathered retrospectively will be very unreliable. For prospective studies, it is essential to describe what is considered *the normal post-operative course* for the operation studied if Grade I complications are to be registered accurately.

Chronic pain and quality of life are important outcome variables for ventral hernia repair. With the EuraHS-QoL score, we propose an evaluation for 3 dimensions. We evaluate pain, restriction of activities and the cosmetic outcome with a numerical rating scale. Loos et al. have found a verbal/numerical rating scale to be more efficient and have a lower failure rate than a visual analogue scale [35]. The EuraHS-QoL score can be used pre- and postoperatively, which will allow investigating the impact of our treatment on the patients’ quality of life. The cosmetic result of ventral hernia repair is an outcome parameter that is missing at this moment in our research, although we think it is important when evaluating different surgical approaches.

In the rapidly growing market of medical devices for abdominal wall surgery, the surgeon has the difficult choice of what product to use in what patient. The innovations are providing us with a plethora of choices. There is no time to acquire high-quality data on all these new medical devices. Many products are on the market with little data on their safety and efficacy [36]. There is need for quality control on the implants we use during abdominal wall surgery. Medical devices need a CE mark to be used in the European Union member countries [37]. A CE mark does not guarantee that the medical device has shown to perform safely and efficiently in humans. *A CE certificate is not a quality mark of the devices’ function, but of the quality of their manufacturing!* A system of post-market surveillance is mandatory in the interest of our patients. The European Union is currently also very much involved in these questions of post-market surveillance as was discussed during a “High Level Health Conference” in Brussels on 22 March 2011 [38]. The Council of the European Union adopted on 6 June 2011 in Luxembourg, conclusions on innovation in the medical device sector which are very much in line with our EuraHS project. Our platform will be a good instrument to acquire data concerning post-marketing surveillance.

In conclusion, we express our hope that the EuraHS database will increase the quality and the quantity of outcome reports in repair of ventral hernias. As of 7 June 2012, the platform will be online and will be presented to the surgical community during a EuraHS Launch Symposium in Brussels.

## Footnotes

1 At the initiative of the first author the EuraHS working group was formed during the board meeting of the European Hernia Society at the 4th International Hernia Congress in Berlin on September 10th 2009. The members of the EuraHS working group were either board members or others EHS members known for their interest in hernia classifications and registries. The board accepted the European internationally balanced composition of the working group. The working group members are the co-authors of this publication. The EuraHS working group meetings were: Malmö, Sweden on November 28th 2009; Gdansk, Poland on February 6th 2010; Amsterdam, The Netherlands on September 4th 2010; Ghent, Belgium on May 13th 2011 and Gdansk, Poland on September 23rd 2011.

2 A dialog box (or dialogue box) is a type of window used to enable reciprocal communication or "dialogue" between a computer and its user (Wikipedia).
